# Tick-Borne Encephalitis in Auvergne-Rhône-Alpes Region, France, 2017–2018

**DOI:** 10.3201/eid2510.181923

**Published:** 2019-10

**Authors:** Elisabeth Botelho-Nevers, Amandine Gagneux-Brunon, Aurelie Velay, Mathilde Guerbois-Galla, Gilda Grard, Claire Bretagne, Alexandra Mailles, Paul O. Verhoeven, Bruno Pozzetto, Sylvie Gonzalo, Samira Fafi-Kremer, Isabelle Leparc-Goffart, Sylvie Pillet

**Affiliations:** University Hospital of Saint-Etienne, Saint-Etienne, France (E. Botelho-Nevers, A. Gagneux-Brunon, P.O. Verhoeven, B. Pozzetto, S. Gonzalo, S. Pillet);; Strasbourg University, Strasbourg, France (A. Velay, S. Fafi-Kremer);; Aix-Marseille University, Marseille, France (M. Guerbois-Galla, G. Grard, I. Leparc-Goffart);; Antoine Béclère Hospital, Clamart, France (C. Bretagne);; French National Public Health Agency, Saint-Maurice, France (A. Mailles)

**Keywords:** emergence, France, tick-borne encephalitis virus, tick-borne encephalitis, TBE, viruses, vector-borne infections

## Abstract

Three autochthonous cases of tick-borne encephalitis (TBE) acquired in rural areas of France where Lyme borreliosis, but not TBE, is endemic highlight the emergence of TBE in new areas. For patients with neurologic involvement who have been in regions where *Ixodes* ticks circulate, clinicians should test for TBE virus and other tickborne viruses.

Tick-borne encephalitis (TBE) is a zoonotic disease caused by tick-borne encephalitis virus (TBEV), a flavivirus transmitted to humans by the bite of an infected tick (*1*) and usually acquired during outdoor activities in forest regions. Among the different TBEV subtypes (*2*), the European subtype is transmitted by *Ixodes ricinus* ticks (*1*). In France, TBEV infection is predominant in the northeastern part of the country, notably in the Alsace-Lorraine region, where the number of reported cases recently increased (*3*–*5*). We report 3 autochthonous cases of TBE acquired during the 2017 and 2018 summer seasons in 2 central rural areas of France not previously known to be places of TBEV circulation: Loire (2 cases) and Haute Loire (1 case), located in the Auvergne-Rhône-Alpes region (Tables 1, 2; Figure). The 3 patients provided informed consent to participate in the study.

**Table 1:** Characteristics of 3 case-patients with tick-borne encephalitis acquired in the Auvergne-Rhône-Alpes region of France, 2017–2018*

Characteristic	**Case-patient 1**	**Case-patient 2**	**Case-patient 3**
Medical history	Myelofibrosis associated with a JAK 2 mutation, treated with hydroxicarbamide	None	Zoster Bell palsy in 1990, arterial hypertension, obesity (BMI 34 kg/m^2^)
Outdoor activity			
Date/duration	2017 Jun 2/2 d	2017 Aug 13–19	All year
Location	Allègre region (43270, Haute Loire)	Montarcher forest (42380, Loire)	Saint-Bonnet-le-Courreau (42940, Loire)
Type	Hiking for 10 km	Hiking, camping	Farming
Tick exposure	3 nonidentified insect bites on legs and left arm (no eschar, slight erythema at localizations of bites) while hiking	1 tick bite; tick removed 48 h later	Yes, frequent
Clinical manifestations			
Date of symptom onset	2017 Jun 17	2017 Aug 30	2018 Jul 21
Main clinical signs	Headache, left cervicobrachial neuralgia, asthenia, delayed persistent fever (>38.5°C)	Low-grade fever (38.5°C), headache, cervical pain, nausea, vomiting	Dizziness, headache, fever (38.4°C), unable to lift right shoulder
Physical findings	No abnormality	Neck stiffness	Proximal deficit in right arm; 3 days later, light deficit in right leg, inability to walk because of motor deficit and dizziness
Encephalitis	No	No	Yes
Radiologic findings	Unremarkable cerebral CT scan	None	Unremarkable cerebral CT scan and cerebral MRI
Biological parameters			
CSF analysis	2017 Jun 23	2017 Sep 2	2018 Jul 21 (first one)
Leukocytes, cells/mm^3^	5	62 (50% PMNs)	195 (88% lymphocytes)
Erythrocytes, cells/mm^3^	2	1	51
Proteinorachia, g/L	0.67	0.48	0.77
Glycorachia/glycemia, mmol/L	2.98/5.8	3.4/5.6	3.18/5.68
Etiologic investigations	Absence of HSV, VZV, or enterovirus by PCR or RT-PCR; presence of TBEV IgM	Absence of enterovirus by RT-PCR; presence of TBEV IgM	Absence of HSV, VZV, or enterovirus by PCR or RT-PCR; presence of *Borrelia burgdorferi* IgG in CSF; Reiber index <2; presence of TBEV IgM
Blood analyses	Blood serology negative for *Mycoplasma pneumonia*, *Bartonella henselae*, *Coxiella burnetii*, *Legionella pneumophila*, HIV, hepatitis B and C viruses, *B. burgdorferi* (both in serum and CSF); positive for cytomegalovirus, Epstein-Barr virus, *Toxoplasma gondii*, and *Chlamydia pneumophila*, revealed past immunization	None	Blood serology for *B. burgdorferi* IgG >0; blood serology negative for *M. pneumonia*, *B. henselae*, *C. burnetii*, *L. pneumophila*, HIV, hepatitis B and C viruses
Treatment	2017 Jun 17: paracetamol; 2017 Jun 19: ceftriaxone 1 g/d + levofloxacin 1 g/d; 2017 Jun 23: treatment stopped	2017 Feb 17: ceftriaxone 100 mg/kg/d; 2017 Sep 4: ceftriaxone stopped, switched to doxycycline 200 mg/d	2018 Jul 21: acyclovir 3,000 mg/d amoxicillin 12 g/d; 2018 Jul 27: acyclovir stopped, amoxicillin switched to ceftriaxone 2 g/d for 14 d
Outcome	Headache and asthenia waned progressively, fever disappeared; discharged 2017 Jun 29	Discharge 2017 Sep 4	Discharged 2018 Aug 17 to rehabilitation center because of persistent dizziness and motor deficit in right arm and leg
Follow-up	Consultation 2017 Jul 27; patient felt good, no headache or fever	Consultation 2017 Sep 18: complete recovery	Consultation 2018 Sep 19; patient able to walk alone but always with a slight motor deficit of right arm and leg and dizziness
Sequelae	No	No	Yes

*Case-patient 1, 76-year-old man; case-patient 2, 8-year-old boy; case-patient 3, 66-year-old woman. No patients had been vaccinated against arboviruses. BMI, body mass index; CSF, cerebrospinal fluid; CT, computed tomography; HSV, herpes simplex virus; MRI, magnetic resonance imaging; PMN, polymorphonuclear cell; RT-PCR, reverse transcription PCR; TBEV, tick-borne encephalitis virus; VZV, varicella zoster virus.

**Table 2:** Results of serologic testing for arboviruses and Lyme disease for 3 patients with tick-borne encephalitis, Loire and Haute-Loire, Auvergne-Rhône-Alpes Region, France, 2017–2018*

Case no., sample	Days after clinical onset	TBEV			DENV			CHIKV			ZIKV			WNV			TOSV			*Borrelia burgdorferi*	
		IgM	IgG		IgM	IgG		IgM	IgG		IgM	IgG		IgM	IgG		IgM	IgG		IgM	IgG
1																					
CSF	16	**4.3**	**3.22**		1	1.12		<1	<1		<1	1		1	1.14		ND	ND			
Serum	19	**6.6**	2.94		1.16	1.24		<1	1.1		ND	ND		<1	1.24		<1	1		Neg	Neg
Serum	48	**7.1**	**10.6**		1.11	1.10		1	1		ND	ND		ND	ND		ND	ND			
2†																					
CSF	15	**53.4**	**748.62**		ND	ND		ND	ND		ND	ND		ND	ND		ND	ND		Neg	Neg
3																					
CSF	2	2.84	1.74		1.09	<1		<1	<1		<1	<1		<1	1		ND	ND			Pos‡
Serum	ND	ND	ND		ND	ND		ND	ND		ND	ND		ND	ND		ND	ND		Neg	Pos
CSF	10	**7.26**	**6.41**		1.05	1.41		<1	1.02		<1	<1		<1	1.39		<1	<1			Pos
Serum‡	10	2.34	**3.02**		<1	<1		<1	<1		<1	<1		<1	<1		<1	<1		Neg	Pos
Serum	61	**4.02**	**5.19**		1.02	2.10		1.02	<1		<1	1		1.06	**3.70**		ND	ND		ND	ND

*For cases 1 and 3, the results of ELISA serologic testing performed at the National Reference Centre for Arboviruses (Marseille, France) are expressed as the ratio between the optical density obtained with each viral antigen and the optical density with the control antigen. A ratio of <2.5 is considered a negative result; a ratio of 2.5–3.0 is considered an undetermined result; a ratio of >3 is considered a positive result. Lyme borreliosis serology was performed with Vidas immunoassays (bioMérieux, https://www.biomerieux.com). Serum specimens reacting for *Borrelia burgdorferi* IgG were also tested with Western blot; reactivity was observed for all cases with the lipoprotein VIse (a variable surface antigen of *B. burgdorferi*), and 17-, 39-, and 83-KDa proteins. Serologic testing for case-patient 2 was performed at the Laboratory of Virology at Strasbourg (Strasbourg, France) by using commercial assays (SERION ELISA classic TBE Virus IgG/IgM; https://www.virion-serion.de/en) and were interpreted according to the manufacturer’s instructions; results are expressed as U/mL, and positive cutoff values were 15 U/mL for TBEV IgM and 150 U/mL for TBEV IgG. Lyme borreliosis serology was performed with Enzygnost immunoassays (Siemens, https://www.siemens-healthineers.com). Boldface indicates positive results. CHIKV, chikungunya virus; CSF, cerebrospinal fluid; DENV, dengue virus; ND, not done; TBEV, tick-borne encephalitis virus; TOSV, Toscana virus; WNV, West Nile virus; ZIKV, Zika virus.  †Serologic testing could not be performed on serum sample. ‡The Reiber index showed a CSF/serum IgG ratio of 1.03 (i.e., an equivocal result of <2).

## The Cases

In June 2017, a 76-year-old immunosuppressed man (case-patient 1) was admitted to the emergency department of a local hospital for headache and cervicobrachial neuralgia. He reported having been hiking in Haute Loire. After symptom persistence and onset of fever over the next 48 hours, he was transferred to the University Hospital of Saint-Etienne (Saint-Etienne, France). Because clinical presentation was unusual and no etiology was determined, serum and cerebrospinal fluid (CSF) samples were sent to the National Reference Centre for Arboviruses (Marseille, France). ELISA detected IgM against TBEV in both fluids. During follow-up testing, serum TBEV IgM and IgG titers increased. The patient’s outcome was favorable, without sequelae.

In September 2017, an 8-year-old boy (case-patient 2) was admitted to the emergency department of the University Hospital of Clamart, near Paris, France, for meningeal syndrome. Two weeks earlier, he had stayed for vacation in the Loire countryside, where he experienced a tick bite. Lumbar puncture results revealed meningitis. A CSF sample was sent to the Borrelia National Reference Centre at the University Hospital of Strasbourg (Strasbourg, France) to rule out Lyme disease; the CSF sample was then transferred to the virology laboratory of the same hospital, where it was positive for TBEV IgM and IgG. The patient recovered without sequelae.

In July 2018, a 66-year-old female farmer (case-patient 3) in Loire, who had been bitten by ticks while working, was first admitted to the emergency department of a local hospital for meningoencephalitis. She was then transferred to the University Hospital of Saint-Etienne. Serologic testing for Lyme disease was positive by ELISA and Western blot for IgG, with no IgM in serum and CSF specimens; Reiber index was <2. Because no alternative etiology was initially found, the patient received treatment for neuroborreliosis. A second lumbar puncture performed 1 week after admission revealed elevated leukocytes (29 cells/mm^3^; 97% lymphocytes), elevated erythrocytes (136 cells/mm^3^), elevated protein level (0.72 g/L), and glucose level within reference range (3.02 mmol/L). Serum and CSF specimens were positive for TBEV IgM and IgG, which ruled out neuroborreliosis and led to discontinuation of antimicrobial therapy. Three months after the acute episode, the patient still experienced dizziness and slight motor deficits in her right arm and leg.

## Conclusions

These 3 cases of TBE occurred in 2 close areas of the Auvergne-Rhône-Alpes region, France, not previously identified as places of TBEV circulation. TBEV emergence in new regions of Europe has recently been described (*5*–*7*). In France, in addition to the Alsace-Lorraine region (*3*), sporadic cases were reported in other rural and forested regions, such as the Alpine region (*2*) (Figure), suggesting that circulation of TBEV in France is wider than previously thought. The increasing number and geographic extension of cases can be related to climate changes, importation of infected ticks by animal migration/transportation, modification of lifestyle with travel and exposure to infected ticks by outdoor activities, and more systematic serologic testing for this agent (*3*,*6*).

**Figure:**
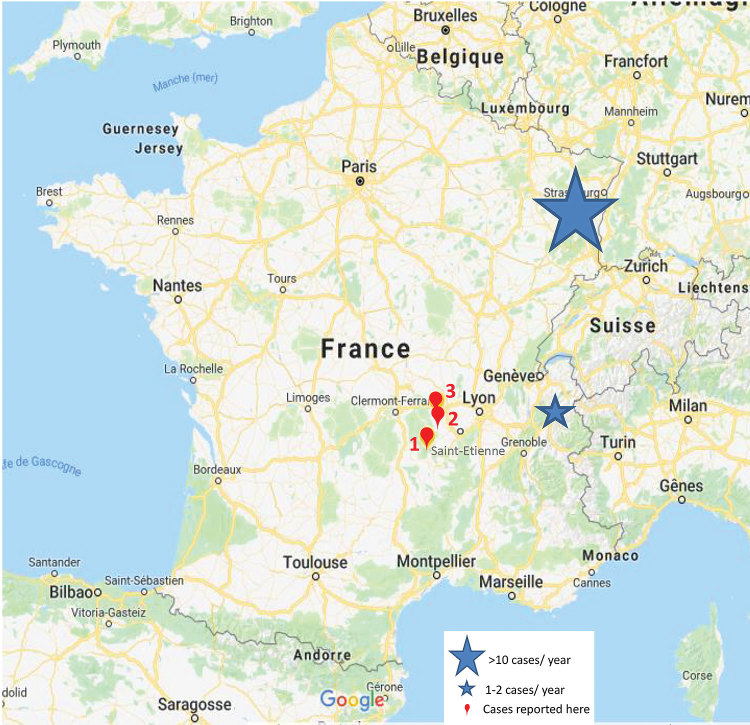
Areas of the Auvergne-Rhône-Alpes region of France visited by 2 patients and inhabited by 1 patient who acquired tick-borne encephalitis during 2017–2018. Red flags and text indicate locations and case-patient numbers.

Of note, *I. ricinus* ticks, the vectors of TBEV in western Europe, are also the vectors of *Borrelia burgdorferi*. Co-circulation of both pathogens could then occur in the same area as reported in Alsace, as suggested by the cases reported here and elsewhere (*8*); Haute Loire and Loire are places with high incidence of Lyme borreliosis (*9*). The prevalence of TBEV infection in ticks has been reported to be low in Europe, notably in France (*10*,*11*). Performance of diagnostic tools for detecting TBEV infection in sentinel animals seems to be better than testing ticks to estimate TBEV circulation in regions where *I. ricinus* ticks are present (*10*).

In Europe, transmission of TBEV occurs mainly from spring through early autumn (*1*,*4*), as found for the 3 cases reported here; this seasonality corresponds to suitable temperatures and humidity required for tick activity (*1*). The viral cycle involves animal reservoirs, mainly rodents and deer; humans are incidental hosts. The most common mode of TBEV transmission is the bite of an infected tick; however, transmission by consumption of unpasteurized milk from infected mammals (goats, sheep, cows) is also suspected (*6*,*12*,*13*). For case-patient 1, transmission probably occurred through a tick bite, even if no tick was seen by the patient; the patient denied consumption of at-risk food. For case-patient 2, a tick was attached to the patient some days before symptom onset. For case-patient 3, transmission by a tick bite is also likely because the patient reported having frequently been bitten by ticks during her professional activity.

In TBE-endemic areas of Europe, TBEV infection is a public health concern; in several countries, vaccination is recommended. Indeed, even if most of the infections caused by the TBEV European subtype are clinically inapparent or only mildly symptomatic, the mortality rate is estimated to be ≈1%, and incomplete recovery with long-term neurologic sequelae is reported for 26%– 46% of cases (*6*). Case-patient 1 exhibited atypical and mild symptoms, consisting of headache and fever without neurologic sequelae. Case-patient 2 exhibited the classical biphasic form of the disease with meningitis that evolved favorably. Case-patient 3 had more severe meningoencephalitis with sequelae.

For case-patients 1 and 3, the profile of acute infection suggested by ELISA was confirmed by plaque-reduction neutralization testing (Table 2). This testing could not be done for case-patient 2 (the young boy) because of insufficient CSF volume. For most cases, even if TBEV can be detected by culture or reverse transcription PCR of serum during early infection when the symptoms are not evocative of TBE (*6*,*14*), a TBE diagnosis is made by serologic testing only. Considering the clinical manifestations, the exposure to tick bites, and serologic results according to the guidelines of the European Academy of Neurology (https://www.ean.org), the 3 cases that we report can be classified as confirmed TBEV infection (*4*).

These cases of TBEV infection highlight the emergence of TBEV in rural and forested areas of France and underline that TBEV infection is probably underdiagnosed in France. Because TBEV and *B. burgdorferi* are carried by the same vector, clinicians with patients who have been bitten by ticks should consider and investigate infection with both pathogens, as well as other tickborne viruses, such as Powassan virus in North America (*15*). To better document the circulation of these viruses, epidemiologic studies are needed. When diagnosing acute neurologic involvement in patients who stayed in regions where *Ixodes* ticks circulate, serologic testing for TBEV and other tickborne viruses should be performed, according to geographic regions. This testing could improve diagnosis of these infections and, according to the evolution of the epidemiology, might be used to modify the TBEV vaccination policy in areas with high TBE incidence.
